# Mental Health Experiences and Needs of Women With Congenital Heart Disease in Pregnancy and Postpartum: A Qualitative Participatory Study

**DOI:** 10.1111/hex.70676

**Published:** 2026-05-31

**Authors:** Caroline J. Huxley, Nigel E. Drury, Anna C. Lavis, Donna Giles, Jo Harrison, Katharina Kaplan, Joanne Lloyd, Tracey Nugent, Charlene Powell, Pameljit Rama, Debbie Rigby, Heather Still, Imogen Tacey‐Green, Karin Eli

**Affiliations:** ^1^ Warwick Medical School University of Warwick Coventry UK; ^2^ Department of Paediatric Cardiac Surgery Birmingham Women's and Children's NHS Foundation Trust Birmingham UK; ^3^ Department of Cardiovascular Sciences University of Birmingham Birmingham UK; ^4^ Department of Applied Health Sciences University of Birmingham Birmingham UK; ^5^ Institute for Mental Health University of Birmingham Birmingham UK; ^6^ Lived Experience Participant

**Keywords:** adult congenital heart disease, mental health, participatory methods, postpartum, pregnancy, qualitative research

## Abstract

**Background and Aim:**

In the United Kingdom, mental health and maternal health are now key priorities for research among adults with congenital heart disease. However, worldwide, research on the mental health of pregnant and postpartum women with congenital heart disease is scant, and no UK‐based participatory studies on the topic have been conducted. Our research aim is to explore the mental health experiences and needs of UK women with lived experiences of pregnancy and congenital heart disease.

**Methods:**

We conducted a qualitative participatory study, in which women with lived experiences of congenital heart disease, pregnancy, and birth participated in a series of semi‐structured online group discussions focused on their mental health experiences and needs and on co‐designing future research on the topic. Discussions were recorded and automatically transcribed; transcripts were analysed using inductive thematic analysis.

**Results:**

Eleven women with a range of congenital heart disease diagnoses and experiences took part. Five themes were developed through the analysis: women with congenital heart disease (1) feel they have very little control over preconception‐, pregnancy‐, and birth‐planning; (2) experience anxiety around pregnancy and childbirth; (3) feel that experiencing a high risk, complex pregnancy can be isolating; (4) experience complex relationships with healthcare professionals during pregnancy; and (5) need more postpartum support. A thread running through the first four themes is the need for additional support during the pregnancy journey, in both healthcare and social contexts.

**Conclusion:**

Distress and anxiety were common experiences during preconception, pregnancy, and the postnatal period. These experiences were exacerbated by wider relational and systemic stressors, namely, social isolation, traumatic healthcare experiences, and lack of psychological support during pregnancy and postpartum. Future research should investigate service gaps and points of intervention for improved cardiac maternity care, as well as explore the potential of peer support programmes to reduce the social isolation experienced by women with congenital heart disease.

**Patient or Public Contribution:**

The study followed a participatory design, whereby lived experience participants co‐produced the research with the study team. The lived experience participants took part in a series of four small‐group meetings, focused on sharing experiences and critically reviewing a draft research agenda and questionnaire, developed by the research team based on the groups' shared experiences. Following these meetings, the lived experience participants provided feedback on our analysis and contributed to this manuscript as co‐authors.

## 1 Introduction

While it is known that adults with congenital heart disease (CHD) have high rates of depression and anxiety [1, 2], and that women with CHD often experience physiologically challenging pregnancies [3], research that brings together mental and maternal health in CHD is scarce. Such research is increasingly crucial: with surgical and medical advances, 97% of children born with CHD are now expected to reach adulthood, leading to a steadily growing adult population [4].

Three systematic reviews have explored the antenatal and postnatal experiences of women with acquired or congenital cardiac conditions [5, 6, 7]. These reviews included a small number of studies (maximum 13), predominantly from the US, Canada, Scandinavia, and Australia. They identified the anxiety that women experience both ante‐ and postnatally, their need to be heard within healthcare settings, their sense of isolation, and the importance of social support. Two studies, based in Australia, have examined peripartum mental health among women with acquired, congenital, or genetic heart disease, with a minority of participants having CHD; these studies highlighted the distress and reduced quality of life the participants faced [8, 9]. Similarly, a quantitative study, from Germany, found high rates of depression and anxiety among women with CHD during the peripartum period [10]. One qualitative study, from Italy, has specifically explored emotional facets of pregnancy among women with CHD, highlighting a complex interaction of fear and empowerment attached to becoming a mother [11, 12]. Another qualitative study, from Germany, found that, among young women with CHD, pregnancy could be a pathway to empowerment and meaning; however, for both men and women who participated in that study, the desire to have children was entangled with fears about their own health and about their children's futures – as potential young orphans, or as living with CHD themselves – with some deciding not to reproduce [13, 14]. Along similar lines, a US‐based qualitative study found that while women with CHD felt motherhood afforded a sense of meaning and social belonging, some experienced health‐related fears that affected their decision‐making about becoming or remaining pregnant [15]. A qualitative study from Sweden, which included both male and female parents with CHD, found that parenthood accentuated feelings of vulnerability, suggesting that healthcare services should attend to this population's emotional needs postnatally [16]. In terms of interactions with healthcare services, one study from Jordan found that pregnant women with CHD had a broad spectrum of needs but faced challenges in seeking healthcare [17]. Research from Sweden identified that regular follow‐ups made pregnant women with CHD feel safe but did not remove their anxieties about their own and their baby's health [18].

In the UK, an estimated 125,000 women are living with CHD, most of whom are of reproductive age [19]. In any given year, 0.8% of pregnant women have CHD [20], translating into approximately 6,600 pregnant women in England and Wales [21]. One UK‐based mixed methods study has explored the experiences of women with CHD who received care within multidisciplinary care models, and the clinicians' experiences of delivering care [22]. The authors found variation in multidisciplinary care provision, which depended on local cardiology and obstetric specialists having the relevant expertise and interest. The qualitative findings highlighted that women became anxious and felt unsafe when clinicians tried to ensure they experienced a ‘normal’ pregnancy, which clinicians considered to be positive and desirable [22]. However, the focus of the study was experiences of healthcare, not the mental health experiences and needs of women with CHD in the antenatal and postnatal periods.

Attending to the healthcare needs of adults living with CHD is of increasing importance to the National Health Service (NHS) in the UK. The recent James Lind Alliance (JLA) Priority Setting Partnership in CHD [23] identified both mental health and maternal health as Top 10 national priorities for research in adult congenital heart disease (ACHD):

#3 – What is the impact of living with CHD on mental health in adults and how can this be improved through access to psychological support and other therapies?

#5 – What are the risks and limitations associated with pregnancy, childbirth, and motherhood for women with CHD, and what information and support is available?

In 2022, our team conducted an impact project focused on NHS improvements needed in the congenital cardiac maternity services in Birmingham. This evidenced the urgent need for UK‐based research that focuses on the mental health experiences and needs of women with CHD during pregnancy and postpartum [19]. However, to date, studies focused on the mental health of women with lived experiences of pregnancy and CHD have not been conducted in the UK, and no study has explored the experiences of women with CHD throughout the pregnancy journey: from preconception to the extended postpartum period. Furthermore, no study has included women with lived experiences of CHD and pregnancy as co‐producers of the research.

In this study, we aim to explore the mental health experiences and needs of UK women with lived experiences of pregnancy and congenital heart disease. We prioritise the views, values, and insights of experts‐by‐experience, using a participatory approach. Through this research, we develop the first UK‐based qualitative evidence on the topic, thereby underpinning the development of future research.

## 2 Materials and Methods

### 2.1 Design

The study followed a participatory research design, a well‐established approach in applied health research, public health, and the social sciences, whose aim is to engage participants as active stakeholders and experts‐by‐experience, co‐producing the research with the study team [24]. As such, participatory research may enable real‐world change that reflects participants' priorities, needs, and experiences [25].

### 2.2 Recruitment

The British Heart Foundation, Somerville Heart Foundation, and Young at Heart, heart patient advocacy organisations based in the UK, posted the study announcement on social media and e‐newsletters, inviting potential participants to contact the researchers. An invitation to participate was also sent to two lists of patient and public involvement and engagement (PPIE) participants who had taken part in earlier, related projects and expressed interest in continued involvement.

### 2.3 Data Collection

Based on their scheduling preferences, participants were allocated into one of three co‐production groups. Each group took part in a series of four small‐group meetings, focused on: (1) sharing experiences and service needs; (2) critically reviewing a draft research agenda and questionnaire, developed by the research team based on the groups' shared experiences; (3) critically reviewing the revised questionnaire; (4) providing feedback on the project experience, revised research agenda, and findings. The meetings took place from March to June 2024. Session 1, which focused on experience sharing, was facilitated as a focus group. Example questions included: *In your opinion, what are the unique challenges that women with congenital heart disease face in pregnancy, which you think NHS Trusts should be aware of?; How did you feel when you were pregnant? (Physically, emotionally, socially?);* and *If you were to advise adult congenital heart disease and obstetric teams on how to improve their patients' mental health, what would be your top priority recommendation?* Sessions 2, 3, and 4 were co‐design sessions; in each of these sessions, the facilitator shared a PowerPoint presentation focused on the session's topic and used a topic guide to engage participants in a critical co‐design discussion. For example, in Session 2, the facilitator presented PowerPoint slides with a draft research agenda and a draft questionnaire; the participants shared their thoughts about each agenda item and each questionnaire item when these were presented, with the facilitator annotating and editing the slides in real time, on a shared screen. Of note, while sessions 2, 3, and 4 focused on co‐design, participants also chose to engage in experience‐sharing during these sessions, as part of reflecting on design choices and preferences. Following the meetings, participants provided feedback, as co‐authors, on the draft of this manuscript, in which we report on the findings concerning participants' experiences.

Group discussions were facilitated by CJH, a research psychologist, and KE, a medical anthropologist. Both CJH and KE had experienced pregnancy, birth, and community and hospital‐based maternity care in the UK, and reflected on their ‘insider’ and ‘outsider’ positionings throughout the project [26]. The research team also included ACL, a medical anthropologist with a research focus on mental health, and NED, an academic consultant in congenital cardiac surgery. We used tailored topic guides for each set of meetings, whilst also actively encouraging flexibility and participant‐led discussion. All group discussions took place on Microsoft Teams and were automatically recorded and transcribed. Transcripts were then pseudonymised, checked, and corrected by CJH and KE.

Participants also completed a short demographic questionnaire, providing open‐text responses in keeping with the participatory ethos of the study.

### 2.4 Analysis

Demographic data were analysed for frequencies and median (IQR) where appropriate. Participants' descriptions of their CHD were categorised as ‘simple’, ‘moderate’, or ‘severe’ according to anatomical complexity, using the Bethesda disease complexity classification [27].

Transcripts were analysed using thematic analysis [28]. The transcripts were read and coded inductively at both semantic and latent levels. Initial codes were grouped into eight preliminary themes, which were reviewed and refined. As the participants included women who had recently given birth, women with school‐age children, and women with adult children, we were mindful about the temporal context of experiences and potential differences between past and present healthcare practices, taking note of the data's contemporary relevance. Preliminary findings were discussed critically with the co‐production groups, and participants provided feedback on the draft manuscript, allowing us to undertake revisions collaboratively and produce materials that accurately reflect the experiences, views, and priorities of women with lived experiences of pregnancy and CHD.

### 2.5 Ethical Approval

This study was reviewed and approved by the University of Warwick's Biomedical and Scientific Research Ethics Committee. All participants received a participant information sheet during the recruitment process, and all provided verbal informed consent at the beginning of the first discussion group.

## 3 Results

We received 14 expressions of interest. Two potential participants dropped out before the study commenced; one participant only joined half of the meetings and her data were therefore not included in the analysis. A total of 11 women completed the study. Participants were allocated into three co‐production groups. To ensure flexibility, when participants could not meet with their allocated group for a particular session, they joined another group for that session or attended an individual meeting. Of note, one participant attended the two last sessions as one‐on‐one meetings with KE, one group had to split for the final session, attending it in pairs, with CJH facilitating, and in three other cases, sessions 2, 3, and 4 were attended by two participants each.

Participants ranged in age from 30 to 62 years (median 35 years, IQR 7.5). Nine (82%) had been diagnosed with CHD early in life, one during pregnancy and one postpartum. Sample characteristics are provided in Table 1.

**Table 1:** Participant characteristics.

Characteristic	*n* (%)
CHD classification	
Simple	1 (9%)
Moderate	8 (73%)
Severe	2 (18%)
Number of children	
1	7 (64%)
2	3 (27%)
3	1 (9%)
Ethnic background	
White (British/other)	9 (82%)
Mixed (other)	1 (9%)
Asian	1 (9%)
Employment status	
Employed (full/part time)	7 (64%)
Stay‐at‐home parent	3 (27%)
Retired	1 (9%)
Geographic region	
South East (England)	4 (36%)
Midlands (England)	3 (27%)
South West (England)	1 (9%)
North West (England)	1 (9%)
Scotland	1 (9%)
Wales	1 (9%)

Our analysis identified five themes: women with CHD (1) feel they have little control over preconception‐, pregnancy‐, and birth‐planning; (2) experience anxiety around pregnancy and childbirth; (3) feel that experiencing a high risk, complex pregnancy can be isolating; (4) experience complex relationships with healthcare professionals during pregnancy; and (5) need more postpartum support.

The need for additional support throughout the pregnancy journey, in both healthcare and social contexts, is a thread running through the first four themes. We explore this thread within each of these themes, highlighting the specific support needs participants had at different stages and the sources of support they needed or desired.

Additional quotations are provided in the Supporting Material.

**1. Women with CHD feel they have little control over preconception‐, pregnancy‐, and birth‐planning**

Participants described how their heart condition ‘controls so much of your life’ (P7), and this was reflected in their journey to parenthood. Participants felt they had little agency over whether they could start trying to conceive and the timing of their pregnancy: ‘You are dictated to almost into whether you're able to have a kid or not’ (P1). When they reached a stage where they felt ready emotionally, participants described negotiating with their healthcare teams to gain ‘permission’ (P6) to attempt conception. Several had been advised to have children before a certain age (to reduce cardiac risk), or between surgical procedures, which placed pressure on them to conceive at the appropriate time:

…they [said] ‘You'll have to have your children before we do your next surgery.’ So then I felt like an added pressure of not only as a woman are you against the clock, but I'm now against another clock because of my heart condition.

(P5)

Once they became pregnant, participants described having little autonomy due to the high‐risk nature of their pregnancy: ‘I had no control. I had to do what the doctors told me, and I felt very, I don't know, just that my body wasn't my own’ (P2). Participants were told which hospital they should attend, which healthcare professionals they needed to consult, how long they were able to continue working, and what type of birth they would have. Many participants were told outright they would have a caesarean section, while others were given strict time limits for the second stage of labour, leading to feelings of ‘really big pressure… to perform’ (P6).

While participants recognised that healthcare professionals exercised control to maximise their safety, the lack of agency they experienced had a psychological impact. To cope with this, participants noted they needed clear communication and information from their healthcare professionals.

**2. Women with CHD experience anxiety around pregnancy and childbirth**

A theme running through all participants' experiences was that of anxiety around how pregnancy and childbirth would affect their heart, and whether their heart condition would affect the health of their baby. While participants spoke about specific fears and used fear‐related words (e.g., ‘scared’, ‘afraid’), the fear experiences they described were multiple, cumulative, and sustained, thereby resulting in a long‐term, lingering sense of worry and unease, consistent with anxiety.

Several participants had been afraid that, because of their heart condition, they would not be able to have children at all. Once they were pregnant they were afraid ‘of the unknown’ (P5): not knowing how well their heart would cope with pregnancy and childbirth, and whether there would be long‐term health implications. As one participant described it: ‘Am I going to make it or is something going to happen to me when giving birth? […] I was really scared’ (P8). Heart‐related fears, combined with reliance on specialist cardiovascular services, affected participants' everyday lives during pregnancy. For example, one participant described taking a holiday near a specialist ACHD centre, while another did not leave her home city, in case she needed specialist care.

Having experienced one pregnancy, many participants debated whether to attempt a second. The needs of their first child now had to be included in the decision‐making, which added complexity and heightened fears around their own mortality: ‘my biggest fear now […] is actually the fear of leaving my first child’ (P11). A participant who had gone through a subsequent pregnancy reflected on difficult conversations she had about whose health to prioritise in an emergency, hers or the unborn baby's: ‘I always went into my second pregnancy with the thought that I come first because I had [my first child to care for]. And that is really hard’ (P3). Several participants were not prepared to take further risks, and decided not to have more children.

Participants described fears around whether their CHD would affect their baby's health, including whether their body would be strong enough to support a growing baby, what effect medications would have during pregnancy and breastfeeding, and the impact of potentially giving birth early. Participants also worried about whether their baby had inherited their heart condition – a fear which was particularly acute during the first half of the pregnancy, prior to the 20‐week foetal anomaly scan.

**3. Experiencing a high risk, complex pregnancy can be isolating**

Participants described how ‘different’ (P3) and ‘isolated’ (P7) they had felt throughout their lives because of their CHD, and these feelings intensified during pregnancy. Because there was no visible outward indication of their condition, others (within both medical and non‐medical settings) often did not understand their unique experiences and needs: ‘you look like every other woman that's had a baby from the outside. It's not visible that actually you've got a heart condition’ (P3). Moreover, when comparing themselves to pregnant friends and relatives without complex conditions, participants felt isolated in being unable to relax and celebrate their pregnancies. This was compounded by feeling a need to ‘protect’ their family from their own fears:

‘when you're going through pregnancy and afterwards you do very often … seek to protect the people closest to you from how you're actually feeling or coping or what's going on with you because they're probably worried enough as it is’.

(P6)

The sense of isolation was heightened in antenatal classes, where participants attempted to engage with the content, but found themselves alienated and excluded by ‘natural’ birth ideologies:

[their] attitude towards medicalised births and all of that really, really played on my mind, [it made] me feel as if, that I'm sub‐par in some way because I'm not going to have that natural birth that you carry on going on about.

(P4)

All participants expressed a desire for contact with other mothers or pregnant women living with CHD, emphasising the importance of peer support:

I do think there is power in having a group of people who are going through the same thing […] to have somebody rooting for you, who understands completely what it is like to be living with a condition, can only be positive.

(P9)

**4. Pregnant women with CHD experience complex relationships with healthcare professionals**

Overall, participants trusted that their specialist cardiac and obstetric clinicians knew what was best for their physical health. Some participants could access a joint pregnancy service and praised the expertise and reassurance this provided. In contrast, participants described how little support was offered for their mental health. While they did not expect that cardio‐obstetric specialists would provide mental health support, participants noted that signposting to support would have been beneficial:

they were looking after my physical well‐being, but they weren't looking after my mental well‐being and I was really struggling, struggling mentally, and they weren't acknowledging that. […] I sort of tried to say to them that I needed that support in the hope that they could potentially refer on somewhere […] But there was just nothing from the team I was under…

(P5)

Many participants described giving birth as traumatic; this was compounded by previous traumas related to heart surgery and other invasive treatments. One participant advocated that a trauma‐informed care model should be adopted, particularly by CHD specialists:

Because you know, we've been through… all these traumas, most of us having heart surgery and stuff. […] that question has got to be asked throughout the whole journey of, what is this experience? What effect is this experience having on this person because of the trauma that they've suffered in the past?

(P3)

While participants trusted their cardio‐obstetric specialists, they did not express the same trust in other healthcare professionals. Participants described challenging encounters with midwives who performed routine antenatal checks and provided care on postnatal wards, but did not understand the complexities of CHD. For example, one participant described her ‘unrelenting anger’ towards her community midwives for not understanding how to care for her, for promoting a natural birth ideology, and, in one instance, for referring to her as ‘a burden’. She described this suboptimal care during a complex pregnancy as a reflection of ‘ableism’ (P1).

These challenges were particularly pronounced after the birth, when participants had been discharged from specialist teams onto postnatal wards. Participants described feeling ‘ignored’ (P1) and treated without compassion or empathy by midwives who were ‘scared’ (P8) of them and their heart condition. Participants described how frightening it was to find themselves under the management of midwives who did not fully understand their needs, and how upsetting it was ‘to beg for care’ (P1) following a traumatic birth. They suggested that midwives should be trained to have more awareness of how to care for women with complex health conditions.

A lack of continuity of care was cited by several participants. Participants described seeing healthcare professionals in different teams and hospitals during pregnancy and postpartum. This was problematic when teams did not communicate with each other, and when participants did not have the chance to build relationships with them. The lack of continuity meant that participants had to advocate for themselves, repeatedly sharing clinical information with different teams and describing their needs. This could be challenging when participants felt they were not being listened to, particularly within the context of a complex pregnancy:

when I go to my local hospital I, you know, I go armed with all my paperwork that says everything about my condition. Yet every time I go there, it's a fight to get them to listen to me and you know, and then when you're pregnant on top of that as well, you're not only just fighting for yourself, you're fighting for your unborn baby.

(P3)

To advocate effectively, participants had to research their options and prepare for their consultations, which was described as ‘exhausting’ (P1) during a stressful pregnancy and postpartum period.

**5. Women with CHD need more postpartum support**

Participants described how the frequent medical check‐ups they received during pregnancy contrasted with the postpartum period. Once their baby was safely delivered, participants were moved back to their standard cardiac care. However, fears lingered over the lasting impact of their pregnancy, and participants felt that more frequent cardiac check‐ups would have helped to resolve uncertainties around their health:

I had absolutely gold standard care while I was pregnant. I had 15 heart scans, MRI scans, ultrasound scans, every like 2 weeks […] But as soon as I gave birth […] I didn't have any follow‐ups. I was managing my own medication. […] that was scary.

(P11)

Participants also wanted more education about how to manage their health postpartum. Several needed information on the interaction between medications and breastfeeding, but said that cardiac clinicians and midwives/breastfeeding specialists were often unsure how medications would affect breastmilk, leading to mixed messages: ‘in hospital they were giving me the tramadol. When I got home and a midwife found out that's what I was on and that I was breastfeeding she read me the riot act’ (P4).

Finally, the participants described a strong need for psychological support in the postpartum period, to cope with both CHD‐related distress and birth‐related trauma. Participants explained that trauma lingered for years after giving birth, such that psychological support was needed going forward; as one participant described it: ‘I felt a massive guilt because both my children ended up in NICU after being born. […] even now I don't think I've properly dealt with that guilt’ (P5). Moreover, as another participant explained, new motherhood raised anxieties about heart health and longevity:

And then being a new mum, having this heart condition, thinking how long am I going to live for? Am I going to see [my child] get married? These kind of things do enter my mind.

(P2)

However, participants' need for psychological support went largely unanswered, with most saying their ACHD service did not offer them this support.

## 4 Discussion

This participatory study is the first to explore the mental health experiences and needs of women with CHD who have given birth in the UK. Through a series of co‐production discussions, focused on the entire pregnancy journey, we found that women with CHD experience considerable distress – from preconception to the extended postpartum period. These experiences are contextualised within a wider framework of stressors, which include lacking agency over decisions related to the timing of pregnancy and mode of birth, feeling socially isolated due to having a rare condition, encountering unknowledgeable non‐specialist healthcare professionals, having to advocate for oneself in unsupportive healthcare environments, and needing (but not receiving) additional postpartum support.

Within the experiences of distress the participants described, anxiety was pervasive. This aligned with previous studies which found that adults with CHD, including peripartum women, have higher rates of anxiety compared to the non‐CHD population [10, 29]. However, while research in the general ACHD population has highlighted health anxiety, heart‐related anxiety, or anxiety stemming from overprotection early in life [30, 31], our findings highlight pregnancy‐specific fears that extend to preconception decision‐making, encompassing concerns about the baby and ramifications to the woman's family should her health decline. Importantly, while these fears were specific, they were not phasic – participants described these fears as having a sustained influence on their feelings, thoughts, and decision‐making, in a manner consistent with anxiety [32]. Similar fears were expressed by postpartum women with CHD who took part in Italian and Swedish studies [11, 16], and were described as influencing subsequent family planning decisions in a US study [15] and in a German study [14]. This underscores the importance of attending to the peripartum period as raising unique life course anxieties.

While considering or undergoing pregnancy with CHD could be distressing for health and family reasons, this was exacerbated by participants' experiences of feeling socially isolated during the pregnancy journey. Previous research has found that people with CHD often experience distress related to a desire for ‘normalcy’, and that this experience extends from childhood into adulthood [33, 34, 35]. However, our findings highlight that women are not distressed by the lack of ‘normalcy’ as much as they are distressed by feeling excluded from normative pregnancy experiences and discourses. As recent studies have found, women with heart disease – congenital or acquired – experience a sense of social isolation, turning to family, patient organisations, or social media for support [9, 11]. Connecting with peers who have experienced both CHD and pregnancy emerged as important in our study. However, our findings also highlighted the role of healthcare services in exacerbating or reducing social isolation, with participants noting that exclusionary discourses about natural birth, such as those used in antenatal classes, had led them to feel even lonelier in their pregnancy experience.

Alongside social isolation, we found that anxieties arising during the peripartum period were interwoven with systemic stressors the participants encountered. Hutchens et al. found that peripartum women with heart disease in Australia experienced trauma related to both health crises and insensitive clinical communication [9]. In our study, experiences with the healthcare system emerged as key to women's narratives of their mental health during pregnancy, birth, and the extended postpartum period. Encountering non‐specialist healthcare professionals who were ill‐informed about CHD, having to advocate for oneself on labour and postnatal wards, facing offensive and dismissive remarks from healthcare professionals, and at times experiencing suboptimal care have been described by participants as upsetting, frightening, and traumatic. Similar to the participants in Hutchens' study [9], most participants said they were not offered psychological support postpartum.

The study's key contribution is in demonstrating how healthcare systems can influence the levels of distress women with CHD face during the peripartum period. While women with CHD may reach pregnancy with a higher baseline level of distress – due to past healthcare experiences, an ongoing sense of social difference, or fears about their health or future – adverse healthcare experiences amplify these existing feelings at a time of great vulnerability. Indeed, the participants' lived experience narratives highlight that healthcare experiences and peripartum distress are inextricably linked, with poor clinical communication and lack of joined‐up care leading to lingering traumatic memories, even years after giving birth.

The unmet need for additional support, particularly psychosocial support, was an overarching issue that permeated the participants' pregnancy and postpartum journeys. Based on the study's findings, we suggest a key area for change is increasing psychology provision within ACHD services. The 2016 NHS England Congenital Heart Disease Standards & Specifications require each ACHD Level 1 centre to ‘employ a minimum of two WTE [whole time equivalent] practitioner psychologists, one of whom will have responsibility for delivering services across the network’ and stipulate that ‘[a]ll patients must be offered access to a Practitioner Psychologist, as appropriate, throughout family planning and pregnancy’ [36]. However, the provision of specialist ACHD psychology services remains lacking across many UK CHD networks. This significantly limits the access that peripartum women with CHD have to appropriate emotional and mental healthcare, equipped to address their specific experiences and concerns. Moreover, the lack of dedicated specialist ACHD psychology services impacts on cardio‐obstetric teams' ability to care for their patients. As explained by Kovacs et al. [37], ACHD clinical psychologists play an important role in training cardiology teams to provide appropriate psychosocial care. Moreover, as case studies from NHS diabetes services have shown, the introduction of specialist psychology care within a dedicated chronic illness service contributes to the upskilling of multidisciplinary teams and to culture shifts in the healthcare environment [38]. We therefore suggest that prioritising psychology provision in ACHD services would be an important step in improving the healthcare experiences and emotional health of peripartum women with CHD.

### 4.1 Limitations

While the study's participatory design was a strength, enabling us to develop a body of knowledge that closely represents the experiences and needs of patient stakeholders, it may have also limited our recruitment, as participation required commitment to a series of discussions. Although study participants represented a wide range of ages and CHD experiences, the sample was predominantly white, and as such our findings may not have captured experiences encountered by those from minority ethnic backgrounds. Finally, we note that the extent to which participants were able to engage with one another and build on each other's insights was limited by variations in attendance – with pairs of participants attending five of the co‐design sessions, and with one participant attending the penultimate and final co‐design sessions as one‐on‐one meetings.

## 5 Conclusion

In this participatory study with women with CHD who had given birth in the UK, we found that while distress and anxiety were common during preconception, pregnancy, and the postnatal period, these experiences were contextualised within wider relational and systemic stressors. Key among these stressors were social isolation, traumatic healthcare experiences, and lack of psychological support. We recommend that future research focus on investigating service gaps and points of intervention for improved cardiac maternity care, as well as designing and evaluating peer support programmes to reduce the social isolation women with CHD experience.

## Author Contributions

**Caroline J. Huxley:** writing – original draft, writing – review and editing, formal analysis, investigation, data curation, project administration, methodology. **Nigel E. Drury:** conceptualization, funding acquisition, formal analysis, writing – review and editing, methodology. **Anna C. Lavis:** conceptualization, funding acquisition, writing – review and editing, formal analysis, methodology. **Donna Giles:** investigation, writing – review and editing **Jo Harrison:** investigation, writing – review and editing. **Katharina Kaplan:** investigation, writing – review and editing. **Joanne Lloyd:** investigation, writing – review and editing. **Tracey Nugent:** investigation, writing – review and editing. **Charlene Powell:** investigation, writing – review and editing. **Pameljit Rama:** investigation, writing – review and editing. **Debbie Rigby:** investigation, writing – review and editing. **Heather Still:** investigation, writing – review and editing. **Imogen Tacey‐Green:** investigation, writing – review and editing. **Karin Eli:** conceptualization, investigation, funding acquisition, writing – original draft, writing – review and editing, methodology, formal analysis, supervision, project administration, data curation.

## Ethics Statement

This study was reviewed and approved by the University of Warwick's Biomedical and Scientific Research Ethics Committee (BSREC 24/23‐34). All participants received a participant information sheet during the recruitment process, and all provided verbal informed consent at the beginning of the first discussion group.

## Conflicts of Interest

The authors declare no conflicts of interest.

## Supporting information

Supporting File.

## Data Availability

To maintain participant confidentiality, the data collected through this study are not suitable for sharing beyond what is included in the manuscript and supplementary material.
