# Supplementary material for: Mental Health Experiences and Needs of Women With Congenital Heart Disease in Pregnancy and Postpartum: A Qualitative Participatory Study
Source: Health Expect. 2026 May 31;29(3):e70676. doi: 10.1111/hex.70676 (PMC13239756; doi:10.1111/hex.70676)
Supplement: Supplementary file 1 — Supporting File. [file HEX-29-e70676-s001.pdf]

Supplementary material – additional quotes supporting our analysis

1. Women with CHD feel they have little control over preconception-, pregnancy-, and birth-planning

Participants felt they had little agency over whether they could start trying to conceive and the timing of their pregnancy: *“I sort of felt like I had to ask permission and well, am I OK to go ahead and have a [baby]? Yeah. You're putting your decisions in someone else's hands. It should be you and your partner that's making those decisions, not a consultant”* (P5).

Participants were told which hospital they should attend, which healthcare professionals they needed to consult, how long they were able to continue working, and what type of birth they would have: *“the minute I was pregnant was ‘right where you can't, you can't choose which hospital you're going to. You have to go to the [Hospital] because they've got a cardiology department’”* (P6).

While participants recognised that healthcare professionals exercised control to maximise their safety, the lack of agency they experienced had a psychological impact:

> *It's that it's that level of control that the healthcare have. And I understand it. I think it's necessary. And I think it's needed, but that does have an impact on us if they, if they, if they say that you have to do this, you have to do that. You have to do that. And it's like it takes control away from us.* (P1)

2. Women with CHD experience anxiety around pregnancy and childbirth

Several participants had been afraid that, because of their heart conditions, they would not be able to have children at all: *“you were told years before that you might not be able to have a child even back in your teens. And that was my experience as well. And I, all the way through, it's like this this thing hanging over you…”* (P6).

Participants described fears around whether their CHD would affect their baby’s health, including what effect medications would have during pregnancy:

> *they did say to me with the warfarin, they said “if you was to become pregnant, now you're under warfarin and you've got your mechanical heart valve, you'd have to come off that warfarin and let us know straight away because it can kill the unborn baby”. […] I thought, you know what? I can't deal with that kind of thing of worrying. Imagine I don't know if I'm pregnant and then it does that?* (P8)

Participants also worried about whether their baby had inherited their heart condition – a fear which was particularly acute during the first half of the pregnancy, prior to the 20-week foetal anomaly scan:

> *I'm anxious of how baby is or how if, if the baby's healthy, if the heart of the baby's healthy […] the added fear of I need to wait till the 12 weeks scan to know that everything is OK. And then I need to wait till the 20 weeks scan to know that the heart is OK, and until we had two checks because I I was just anxious about that and we had a private check.* (P7)

3. Experiencing a high risk, complex pregnancy can be isolating

When comparing themselves to pregnant friends and relatives without complex conditions, participants felt isolated in being unable to relax and celebrate their pregnancies:

> *on one hand, just so you feel so lucky that you're having this opportunity to have a baby because before you even get pregnant, you don't know whether you are going to be well enough to get pregnant and even have a baby. […] But at the same time, you feel this, I suppose jealousy, envy, I don't know, of other people that have got that opportunity to go to the birthing centre and have this lovely, you know, chilled out, wonderful birth without all these people peering at you.* (P3)

All participants expressed a desire for contact with other mothers or pregnant women living with CHD, emphasising the importance of peer support:

> *I think [what] I would have found really useful was being in, being put in touch with women in a similar situation. Other women who have got heart conditions, who are going through pregnancy or have gone through pregnancy. Doesn't have to be the same heart condition as me because as we know, there's hundreds of different conditions. But knowing that, oh, that was a woman [who] has had a heart condition, she's had a birth and she's had X amount of operations. And look, she's had a successful pregnancy. Because it is. I mean, I think pregnancy for some women could be lonely anyway, but pregnancy with a heart condition can be very, very lonely.* (P5)

4. Pregnant women with CHD experience complex relationships with health professionals

Overall, participants trusted that their specialist cardiac and obstetric clinicians knew what was best for their physical health. Some participants could access a joint pregnancy service and praised the expertise and reassurance this provided:

> *they do a Joint pregnancy service for women with congenital heart conditions […] specifically for women with congenital heart conditions who are pregnant, and they knew exactly what they're talking about. […] They were fantastic. They, they never made me feel inadequate or they made me feel amazing.* (P3)

While participants trusted their cardiac/obstetric specialists, they did not express the same trust in other healthcare professionals. Participants described challenging encounters with midwives who performed routine antenatal checks and provided care on post-labour wards, but did not understand the complexities of CHD:

> *what I found was within the unit of the foetal medicine team and the cardiology unit, brilliant, everybody understood, but as soon as you are on a normal ward nobody knew what was going on.* (P9)

Participants suggested that midwives should be trained to have more awareness of how to care for women with complex health conditions: *"I'm not saying that they all need to be special, [that] the midwives need to be specialist in every CHD, but I think it should be on their radar at least. If you're forcing us to go and to have babies in those centres, then that at least they should know more about us"* (P4).

A lack of continuity of care was cited by several participants. Participants described seeing healthcare professionals in different teams and hospitals during pregnancy and postpartum. This was problematic when teams did not communicate with each other, and when participants did not have the chance to build relationships with them:

> *I [had] kind of become my own health professional. When I was going to those [hospitals] and I didn't really fully understand what was going on with me, but I had to tell them, and then they had to then try and relay stuff. […] I got admitted a couple of times to my hospital near me for pregnancy related issues, not heart related, but pregnancy related and unless I told my team in the big hospital they didn't know about it. It was up to me to tell them, and I found that really difficult.* (P4)

5. Women with CHD need more postpartum support

Participants described how the frequent medical check-ups they received during pregnancy contrasted with the postpartum period. Once their baby was safely delivered, participants

were moved back to their standard cardiac care. However, fears lingered over the lasting impact of their pregnancy, and participants felt that more frequent cardiac check-ups would have helped to resolve uncertainties around their health:

> *Better touch points with your cardiologist 'cause they spend the entire nine months telling you about the risks and everything and then all of a sudden it's you have the baby. And I was meant to go back, I think it was something like just over six weeks to see them. And, but something happened and I wasn't checked up on for a while, but it was like, so you're telling me all these things about my heart but now all of a sudden it's not an issue […] you can't tell someone for nine months that there's a risk of this and then all of a sudden it goes away. That's just terrible for your mental health. So definitely postnatal be something that they look at.* (P4)

Participants also wanted more education about how to manage their health postpartum:

> *I think it's a bit of a lack of education about how I should be looking after myself. I'm not, I mean I am sensible. But how I should be looking after myself? What's the best exercises? […] But I think just linking it back to the aftercare, not having the education of how to look after myself, I've kind of just been let out.* (P2)
